# Supplementary material for: Chromothripsis during telomere crisis is independent of NHEJ, and consistent with a replicative origin
Source: Genome Res. 2019 May;29(5):737–49. doi: 10.1101/gr.240705.118 (PMC6499312; doi:10.1101/gr.240705.118)
Supplement: Supplemental Material [file supp_gr.240705.118_Supplemental_file_1.zip › contigs/annotated_contigs/DB73/contig.2.DB73_length_392_mean_cov_5.44897959184.docx]

**DB73_length_392_mean_cov_5.44897959184**

```
AGTTTTTGTATTTTTAGTACAGACGGGGTTTCACAATGTTGGTCAGGCTGGTCTTGAACTCCTAACCTCAAGTGATCCACCCGCCTCGG
 >chr12:93355405-93355556 - E=2e-79
CCTCCCAAAGTGCTGGGATTACAAGGGTGAGCCACCACTCCTGGCCTGTTCATCTTTTTAGA|TGAA|CACAGGGATATATTGCAATTG
                                                                   >chr12:93362202-9336
CTGTTTTCCTTCTTTTCTCCAAGAAATAATATGCTCCATGGGAACAGAGATAGTGTCTTGTTCATTGGGAGCATTCCTAGCACTGGACA
2439 + E=3e-125
TAGTGCCTGAGACTTAAACTGCTCATAACTGTTTCTTTGAAGGAAACGAAAAAGGCAGGGAGGGAGGAAAGGAGGGAGGGAGGCAGGAA

GGAAGGGAGGAAATGAATCAGCATGAGAAGATCAGGGC
```
